# Author response: Effect of temperature changes between neighboring days on acute aortic dissection in non-heating periods

**DOI:** 10.1016/j.lanwpc.2022.100659

**Published:** 2022-12-05

**Authors:** Qingli Zhang, Haidong Kan

**Affiliations:** School of Public Health, Key Lab of Public Health Safety of the Ministry of Education and NHC Key Lab of Health Technology Assessment, Fudan University, Shanghai, China

We are pleased to respond to Noriyuki Yamato and Tatsuya Fujikawa for their comments on our recent study about the effects of temperature changes between neighboring days (TCN) on acute aortic dissection (AAD) in non-heating periods.^1^

Yamato and Fujikawa suggest that it is debatable whether central heating reduces risks of AAD induced by negative TCN, because they think negative TCN does not significantly increase relative AAD risk not only in heating periods in cities with central heating, but also in winter in cities without central heating. First, the role of central heating seems to be over-interpreted by Yamato and Fujikawa. In fact, we just objectively presented the finding that “*central heating may be a modifier for temperature-related AAD risk and burden*”, but not stated “*central heating reduces risks of AAD*”. In the discussion section, we suggested potential protective effect of central heating as one of the possible explanations for the differential effects of TCN on AAD in heating and non-heating period. Although the differential effects might be not only due to central heating, but also related to other factors, the potentially critical role of indoor heating cannot be overlooked. Second, there seems to be some misunderstanding by Yamato and Fujikawa on the effect of TCN in winter. It is not like they stated that “*negative TCN did not significantly increase relative AAD risk”*, negative TCN in fact significantly increases relative AAD risk in the linear function (Table 1), and the exposure-response curve in Figure 4e also presents a marginally significant association in winter in cities without central heating. We acknowledge that in cities without central heating, the association between negative TCN and AAD is weaker in magnitude in winter than in other seasons. The lower risk observed in winter suggests that some personal protective procedures might also play a role in mitigating the harmful effect of negative TCN. Of note, although central heating is not provided in cities without central heating in China, personal protective procedures such as air conditioning and underfloor heating are widely used in winter in these cities.

As Yamato and Fujikawa mentioned, we found there is an association between increased AAD onset and negative TCN in cities with central heating. Exactly because the significant association in non-heating periods and the non-significant association in heating period, we assumed that central heating might modify the association between TCN and AAD onset. Nevertheless, we have never stated that the significant association occurred during non-heating period is an effect of central heating.

Another concern Yamato and Fujikawa raised is whether sudden drops in temperature are associated with increased AAD onset during non-heating seasons. The independent effects of negative TCN on cardiovascular diseases have been widely reported in previous studies.^2^^,^^3^ In our study, we also found negative TCN was independently associated with increased AAD onset in non-heating seasons after adjustment for ambient temperature and other potential confounders. In the discussion section, we speculate that the deleterious effects of sudden drops in temperature on AAD might be attributed to its impacts on the autonomic nervous, and subsequent effects on evaluated blood pressure. Yamato and Fujikawa suggest that it is unclear whether the negative association between temperature and blood pressure also applies during the non-heating season, particularly in summer. They cited a study by Kang et al., which reported that above a certain limit, increased temperature was associated with elevated blood pressure in warm season. However, a large number of previous studies consistently found that low temperature was associated with evaluated blood pressure,^4^ while very few studies have reported a positive association between temperature and blood pressure no matter in summer or not. Besides, the exposure-response curves in most previous studies showed that temperature decrease was associated with increased blood pressure even in high temperature ranges (e.g., higher than 25 °C), which are more likely to occur in summer.^5^^,^^6^ Moreover, in the study by Kang et al., the fact that increased temperature was associated with elevated blood pressure above a certain limit was observed only in a few climatic zones (temperature monsoon zone and temperature continental zone), while in subtropical zone, increased temperature was associated with decreased blood pressure across all temperature ranges.^7^ In our study, however, a large proportion of cases are located in subtropical zone, but not in temperature monsoon zone nor temperature continental zone. Therefore, it is reasonable to assume that temperature drop might increase blood pressure in our study population regardless of whether in summer or not.

We agree with Yamato and Fujikawa that the summer increases in nighttime blood pressure might not be due to temperature. However, summer increases in nighttime blood pressure only present the circadian rhythms of the blood pressure, but not the association between temperature and blood pressure. Our focus is the association between temperature and blood pressure for a person in the same calendar year, month, day of week, and **same hour of day**, thus the circadian rhythms can be automatically controlled.

Therefore, we think TCN is independently associated with AAD onset but not just a confounding factor in non-heating period in the current study. Because TCN might also be a confounding factor in the association between temperature and AAD onset, we have controlled for TCN in the main analyses.

## Declaration of interests

We declare no competing interests.
